# Can AI Detect What Is Not Injected? Evaluation of Lesion Detection in Virtual Contrast-Enhanced Breast MRI Using a Large-Scale AI Model Trained on GBCA-Enhanced Data

**DOI:** 10.3390/tomography12070105

**Published:** 2026-07-16

**Authors:** Shirin Heidarikahkesh, Hannes Schreiter, Aju George, Tri-Thien Nguyen, Dominika Skwierawska, Luise Brock, Dominique Hadler, Michael Uder, Frederik B. Laun, Chris Ehring, Johanna Graber, Lorenz Döppmann, Ihor Horishnyi, Lorenz A. Kapsner, Sabine Ohlmeyer, Andrzej Liebert, Sebastian Bickelhaupt

**Affiliations:** 1 Institute of Radiology, Universitätsklinikum Erlangen, Friedrich-Alexander-Universität Erlangen-Nürnberg (FAU), 91054 Erlangen, Germany; hannes.schreiter@uk-erlangen.de (H.S.); aju.george@uk-erlangen.de (A.G.); tri-thien.nguyen@uk-erlangen.de (T.-T.N.); dominika.skwierawska@uk-erlangen.de (D.S.); luise.brock@uk-erlangen.de (L.B.); dominique.hadler@uk-erlangen.de (D.H.); michael.uder@uk-erlangen.de (M.U.); frederik.laun@uk-erlangen.de (F.B.L.); chrismatthias.ehring@usb.ch (C.E.); johanna.graber@uk-erlangen.de (J.G.); lorenz.doeppmann@uk-erlangen.de (L.D.); igorgorishniy97@gmail.com (I.H.); lorenz.kapsner@uk-erlangen.de (L.A.K.); sabine.ohlmeyer@uk-erlangen.de (S.O.); andrzej.liebert@uk-erlangen.de (A.L.); sebastian.bickelhaupt@uk-erlangen.de (S.B.); 2 Pattern Recognition Lab., Friedrich-Alexander-Universität Erlangen-Nürnberg (FAU), 91054 Erlangen, Germany; 3 Department Artificial Intelligence in Biomedical Engineering, Friedrich-Alexander-Universität Erlangen-Nürnberg (FAU), 91054 Erlangen, Germany; 4 Department Medical Informatics, Friedrich-Alexander-Universität Erlangen-Nürnberg (FAU), 91054 Erlangen, Germany; 5 Institute of Computer Science, Polish Academy of Science, 01-248 Warsaw, Poland

**Keywords:** gadolinium-enhanced MRI, lesion detection, virtual contrast enhancement

## Abstract

Background/Objectives: Artificial intelligence (AI) can support lesion detection in gadolinium-based contrast agent-enhanced (GBCA-enhanced) breast MRI. However, its effectiveness on virtual contrast-enhanced (vCE) images remains unclear. This feasibility study evaluated the publicly available MAMA-MIA nnU-Net model trained on GBCA-enhanced data using an independent cohort of both GBCA-enhanced and vCE breast MRI. Methods: This IRB-approved retrospective study included the publicly available nnU-Net model trained on *n* = 1506 MAMA-MIA breast MRI scans and a cohort of *n* = 2126 in-house 3T breast MRI scans. A generative adversarial network (Pix2Pix-GAN) was developed on *n* = 1870 of the in-house scans and used to generate vCE data on the remaining independent *n* = 256 in-house cases. The MAMA-MIA nnU-net was applied to both GBCA-enhanced (GBCA) and corresponding vCE images. Ground-truth segmentations of malignant lesions served to calculate the Dice score, Hausdorff distance, and lesion dimension differences. Results: The final test set comprised *n* = 250 cases (*n* = 69 malignant, *n* = 181 benign). Lesion detection rates were 91% (*n* = 63/*n* = 69; 95% confidence interval (CI): 82.3–96.0%) for GBCA and 84% (*n* = 58/*n* = 69; 95% CI: 73.7–90.9%) for vCE. Two lesions missed in GBCA were identified by vCE. The Hausdorff distances were similar (GBCA: 6.4 (IQR: 3.2–9.3; 95% CI: 5.2–7.8) mm; vCE: 6.7 (IQR: 3.9–9.7; 95% CI: 5.3–8.0) mm, *p* = 0.564). The Dice scores showed minor differences (GBCA: 0.829 (IQR: 0.723–0.900; 95% CI: 0.786–0.865) vs. vCE: 0.826 (IQR: 0.720–0.857; 95% CI: 0.770–0.836); *p* < 0.001). vCE images had slightly higher non-target tissue segmentation (median 6072 mm^3^ vs. 5754 mm^3^). Conclusions: A GBCA-trained algorithm demonstrated some cross-domain transferability to vCE images, albeit with a reduced case-level sensitivity of 84% (95% CI: 73.7–90.9%) vs. 91% (95% CI: 82.3–96.0%). Based on these preliminary results, further research, including larger cohorts and more diverse datasets, is warranted.

## Simple Summary

Artificial intelligence can help find breast lesions on GBCA-contrast-enhanced MRI, but it is unclear how well models trained on such data perform on so called virtual contrast-enhanced images generated from unenhanced acquisitions. This IRB-approved retrospective feasibility study evaluated the publicly available MAMA-MIA nnU-Net model (trained on *n* = 1506 contrast-enhanced scans) on *n* = 250 in-house cases comprising both contrast-enhanced and matched virtual contrast-enhanced images created by a Pix2Pix-GAN. The detection rate for malignant lesions was 91% (95% CI: 82.3–96.0%) for real contrast and 84% (95% CI: 73.7–90.9%) for virtual contrast-enhanced images with similar overall segmentation accuracy; interestingly, two malignant lesions missed on real contrast-enhanced images by the MAMA-MIA nnU-Net were detected on virtual contrast-enhanced images.

## 1. Introduction

Breast magnetic resonance imaging (MRI) is the most sensitive imaging method for detecting suspicious breast lesions [1,2]. It is routinely performed using a multiparametric protocol that includes the intravenous application of gadolinium-based contrast agents (GBCAs) [3]. As women with dense breast tissue are known to experience reduced sensitivity in X-ray mammography, the European Society of Breast Imaging (EUSOBI) has proposed breast MRI as a potential supplemental imaging method for women with extremely dense breast tissue [4].

However, despite its diagnostic capabilities, breast MRI has not been widely adopted for population-based breast cancer screening programs. Commonly cited reasons include the effort and costs, which are highly directly and indirectly associated with the routine use of GBCAs [5,6,7]. In addition, follow-up results from the Dense Tissue and Early Breast Neoplasm Screening (DENSE) trial [8] indicated that women declining or opting out of the MRI screening mention factors associated with GBCA application in an increasing manner from 13.7% in the second screening round to 20.5% in the third [5].

Whilst GBCA-enhanced breast MRI thus remains the established clinical standard for breast lesion detection and characterization, these findings (amongst others) motivate research into potential complementary imaging strategies which might expand the overall available diagnostic toolbox of breast imaging and might enable the reduction or in some case omission of the application of intravenous contrast agents in specific indications, such as in supplemental screening of women with dense breast tissue [9,10,11,12,13,14]. Among the various techniques under investigation, diffusion-weighted imaging (DWI) has suggested potential for lesion detection in breast cancer screening as an unenhanced breast MRI approach when specifically adapted and is being investigated in ongoing prospective studies (e.g., the Diffusion-Weighted Magnetic Resonance Imaging Screening Trial (DWIST)) [15]. Retrospective analyses have suggested improved image quality of DWI in women with dense breast tissue [16] and that it may help detect early-stage breast cancer [17].

Reading diffusion-weighted images however can be challenging given their markedly different contrast behavior from morpho-anatomically GBCA-enhanced images. Recent advances in deep learning techniques have indicated the potential of using a non-contrast-enhanced MRI protocol to generate images that mimic the characteristics of GBCA-enhanced images, often referred to as virtual contrast-enhanced (vCE) images [18,19,20,21,22,23].

In addition to image generation, artificial intelligence (AI) applications are increasingly being developed to support radiologists in lesion detection tasks. Recently, a multinational consortium published a comprehensive breast MRI dataset (MAMA-MIA) collected across multiple sites, along with a model for breast lesion detection trained using nnU-Net—a self-configuring deep learning approach for image segmentation [24]. The MAMA-MIA dataset and algorithms reflect growing efforts to provide a substantial resource aimed at improving the robustness and generalizability of AI algorithms for lesion detection and characterization in breast MRI. However, given the conceptual developments of vCE breast MRI, it remains unclear whether AI algorithms trained on GBCA-enhanced MRI are (at least partially) generalizable to vCE images or fully collapse due to Out-of-Order Distribution (OOD).

In our technical feasibility study, we compare the performance of the MAMA-MIA-provided nnU-Net, trained on public GBCA-enhanced breast-MRI data, between an independent holdout test set of GBCA-enhanced and generative adversarial network (GAN)-generated vCE breast MR images.

## 2. Materials and Methods

### 2.1. Datasets

This IRB-approved retrospective study is based on two breast MRI datasets comprising a total of *n* = 3632 breast MRI examinations from internal and external studies, including multiple study sites.

External datasets: The MAMA-MIA breast MRI dataset [24] consists of *n* = 1506 MRI examinations of breasts with malignant lesions, sourced from four publicly available collections in The Cancer Imaging Archive (TCIA) [25]: the I-SPY1/ACRIN 6657 trial (I-SPY1) [26], the I-SPY2/ACRIN 6698 trial (I-SPY2) [27], the NACT-Pilot trial (NACT) [28], and Duke-Breast-Cancer-MRI (DUKE) [29]. The dataset is accompanied by expert segmentations of the malignant target lesions for all cases. Further details of the underlying cohorts and MRI protocols are available in [24].

**Figure 1:**
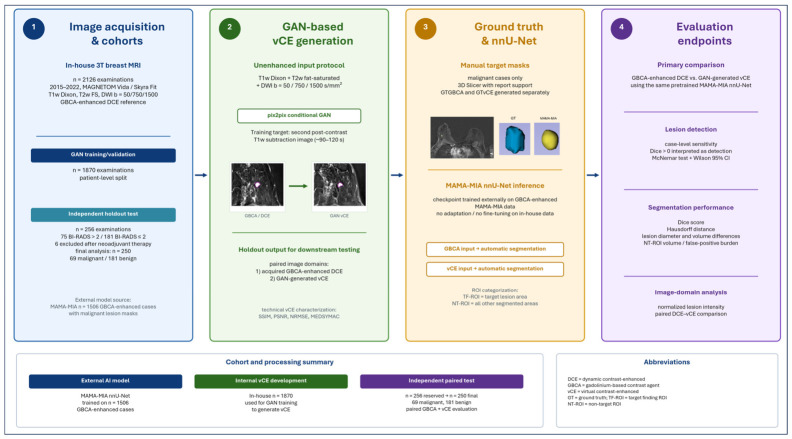
Flowchart depicting the workflow of the pipeline. The in-house dataset, which consisted of *n* = 2126 breast MRI examinations acquired with two scanners MAGNETOM Vida and MAGNETOM Skyra Fit, using T1-weighted, T2-weighted, and diffusion-weighted imaging (DWI) sequences with three b-values (50, 750, and 1500 s/mm^2^). The dataset was split into *n* = 1870 cases used to train a generative adversarial network (GAN) model for generating virtual contrast-enhanced (vCE) T1-weighted, second-time-point subtraction images, and *n* = 256 cases were reserved for independent testing. For testing the MAMA-MIA algorithm, GAN-generated vCE images and their corresponding ground-truth dynamic contrast-enhanced (DCE) second-time-point images were used as inputs. The test set was further stratified by BI-RADS scores: *n* = 75 cases had BI-RADS > 2 (of which six were excluded due to minimal/no residual disease post-therapy), and *n* = 181 cases had BI-RADS ≤ 2. Automated and expert manual segmentations were performed on these subsets. Manual expert-supervised ground-truth segmentations were generated separately on GBCA-enhanced DCE images and GAN-generated vCE images for malignant cases. In addition, it includes the external MAMA-MIA dataset (*n* = 1506 GBCA-enhanced breast MRI examinations), which was used to train the nnU-Net model independently. The pretrained MAMA-MIA nnU-Net checkpoints were then applied without any in-house fine-tuning to both the GBCA-enhanced DCE and GAN-generated vCE images of the independent test cohort. Final evaluation included case-level lesion detection sensitivity, Dice score, Hausdorff distance, lesion diameter and volume differences for target-finding regions of interest, as well as non-target ROI volume analysis.

Internal dataset: Our in-house breast MRI dataset consisted of *n* = 2126 routine breast MRI examinations acquired between 2015 and 2022 as shown in Figure 1.

The inclusion criteria were a clinical indication for breast MRI examination, i.e., preoperative staging, follow-up examinations, high-risk screening, and neoadjuvant therapy response assessment. Examinations were only included if performed on 3T MRI scanners (MAGNETOM Vida and MAGNETOM Skyra Fit, Siemens Healthcare, Erlangen, Germany) using an 18-channel breast coil (Siemens Healthineers, Erlangen, Germany) and a full diagnostic breast-MRI protocol. The protocol had to include T1w Dixon, T2w fat-saturated, and multi-b-value DWI with three b-values (50, 750, 1500 s/mm^2^). Further details are provided in Table 1.

The in-house dataset was split into two parts: a training/validation set (*n* = 1870) and an independent holdout test set (*n* = 256), which was not revealed during the training and validation steps.

The split was performed at the patient level to ensure no patient overlap between the training or validation and the holdout sets for patients with multiple consecutive measurements.

The independent holdout test set was constrained to include equal numbers of acquisitions (*n* = 128) across the two MRI scanner types and between breast MRI examinations with BI-RADS > 2 and BI-RADS ≤ 2 scores, as reported in the original radiological diagnostic reading.

As all datasets originated from clinical routine, women with suspicious findings were undergoing further clarification using ultrasound, stereotactic biopsy, or surgical specimen analysis. Histopathology served as the reference for defining cases with and without breast cancer.

All internal datasets included in the study were previously used to a varying extent in different studies involving the application of AI methods for detection or prediction of artifacts, generation of vCE images and identification of incidental findings—with the manuscripts and their respective topics including the dataset being provided in Appendix A, Table A1.

While prior work from our group established the technical feasibility of GAN-based vCE generation and characterized global image-similarity metrics, none of those studies evaluated whether externally trained, large-scale AI segmentation models generalize to such synthetic images, an inherently out-of-distribution scenario for a model trained exclusively on real GBCA-enhanced data. This is the specific and previously unaddressed question of the present study.

### 2.2. Neural Network Architecture and Generation of Virtual Dynamic Contrast-Enhanced Breast MRI

A neural network was trained to generate vCE breast MRI acquisitions using the in-house training/validation dataset, following the principles of George et al. [19]. In short, a pix2pix conditional GAN was trained using T1w Dixon, T2w fat-saturated, and DWI acquisitions with b-values of 50, 750, and 1500 s/mm^2^ as inputs. The second contrast-enhanced T1w subtraction image (referred to as the GBCA image), acquired approximately 90–120 s after contrast-agent administration, served as the target of the neural network. As a result, the network was trained to generate vCE breast MR images based on unenhanced input acquisitions.

Preprocessing: The in-house dataset was preprocessed for vCE training following the procedures described by George et al. and Schreiter et al. [19,20]. Briefly, the multiparametric input sequences were brought into a common spatial representation using field-of-view resampling, z-dimension alignment, z-score intensity normalization, intensity clamping, and rescaling. The DWI acquisition was used to define the common field of view for the input stack, as it represented the spatial coverage shared by the unenhanced sequences used for vCE generation. The field of view was adjusted based on the DWI acquisitions, with an in-plane resolution of 280 × 448. For through-plane alignment, the input sequences were aligned to the GBCA-enhanced second-time-point subtraction target while retaining the original slice number of the GBCA image, thereby enabling paired slice-wise image-to-image training.

Training: The framework, as described by George et al. [19], performs paired image-to-image translation through adversarial learning between generator and discriminator networks. Both networks were optimized using the Adam optimizer with learning rates of 1 × 10^−4^ for the generator and 1 × 10^−3^ for the discriminator as originally described. Training was performed for 50 epochs on a single NVIDIA A100 (40 GB) GPU.

Technical evaluation of network performance: The performance was evaluated quantitatively. The generated vCE breast images were assessed using the structural similarity index (SSIM) [30], peak signal-to-noise ratio (PSNR), high-frequency error norm (HFEN), median symmetric accuracy (MEDSYMAC), normalized root mean squared error (NRMSE), and mean square error (MSE).

Qualitative assessment of vCE image quality: In addition to quantitative image-similarity metrics, GAN-generated vCE images were qualitatively assessed by a board-certified radiologist with more than 15 years of experience in breast MRI. Image quality was rated on a Likert-like ordinal scale as the following: 1 = insufficient image quality, high level of blurring of morphological structures and/or lesions or high level of artifacts, no diagnostic assessment possible; 2 = low image quality, significant blurring of morphological structures and/or lesions and significant artifacts, diagnostic assessment significantly impaired; 3 = intermediate image quality, partial blurring of morphological structures and/or lesions or artifacts, regionally impeding assessment; 4 = adequate image quality, low level of blurring of morphological structures and/or lesions, low level of artifacts, diagnostic assessment feasible; 5 = high image quality, no blurring or artifacts, diagnostic assessment possible with high confidence.

Exploratory image-domain characterization: To further characterize the image-domain shift between GBCA-enhanced DCE and GAN-generated vCE images, we performed an exploratory lesion-level intensity analysis in the malignant test cases. For each case, median lesion intensity was extracted from the GBCA-enhanced image using the corresponding manual GTGBCA lesion mask and from the vCE image using the corresponding manual GTvCE lesion mask. Because absolute image intensity scaling may differ between acquired and generated images, lesion intensity was normalized to the whole-image intensity distribution using the following formula: normalized lesion intensity = (median lesion intensity—whole-image median intensity)/whole-image interquartile range. Paired DCE-vCE differences in normalized lesion intensity were assessed using the Wilcoxon signed-rank test. Because the vCE images were generated as second-time-point subtraction-like images rather than as a dynamic time series, kinetic enhancement curves or contrast-dynamics parameters were not analyzed. 

### 2.3. Preparation of Data for Evaluation of MAMA-MIA nnU-Net on vCE and GBCA-Enhanced Breast MRI Datasets

The independent holdout test datasets, comprising the vCE and GBCA test sets, were further processed. Using the open-source 3D Slicer software (version 5.03) [31], segmentations were performed on all cases with histopathologically verified malignant masses or NMEs. Segmentations were performed by research assistants (medical students, CE/JG/LD/IH) with 2 years of experience in breast MRI, under the supervision of two board-certified radiologists with >10 years of experience in breast MRI (SB/DH). During segmentation, the clinical diagnostic report was available to the research assistants to support the reliable identification of the target structure. This target structure—defined as the largest solitary lesion or enhancing area in the case of NME, multifocal, or multicentric breast cancer—was contoured along the inner border using the integrated draw function of 3D Slicer, avoiding larger vessels. The process was applied to both vCE and GBCA images, resulting in two target segmentations per breast MRI examination—GTvCE and GTGBCA, respectively.

### 2.4. Automatic Segmentation Generation and Performance Evaluation of MAMA-MIA nnU-Net

The nnU-Net v2.4.2 [32] checkpoints provided in the MAMA-MIA study were used without further adaptation or refinement to automatically segment both the vCE and GBCA images in the independent holdout test sets ensuring complete independence between the nnU-Net training data and the in-house holdout test set. These checkpoints, trained on the external MAMA-MIA dataset, are referred to here as the MAMA-MIA algorithm. Further details of nnU-Net and other details are publicly available at GitHub (https://github.com/LidiaGarrucho/MAMA-MIA, accessed on 25 July 2024). Our institution did not contribute any datasets to the MAMA-MIA datasets in the past.

For examinations with malignant findings, the resulting automatic segmentations were categorized into target-finding regions of interest (TF-ROIs) and non-target regions of interest (NT-ROIs). The TF-ROIs included the primary lesion, which was first identified in the GT_vCE_ and GT_GBCA_ segmentations. A bounding box was drawn around this region and applied to the corresponding location in the segmentations performed by MAMA-MIA nnU-Net, thereby defining the algorithm’s target area. All other segmentations performed by the algorithm in other breast tissue regions were considered NT-ROIs. This process was performed for both vCE and GBCA images, resulting in four types of ROIs—TF-ROI_vCE_, NT-ROI_vCE_, TF-ROI_GBCA_, NT-ROI_GBCA_—which were further analyzed. For examinations without target findings (i.e., benign cases), all automatic segmentations were assigned to the NT-ROI category. This NT-ROI analysis was used to quantify the false-positive segmentation burden of the model at the same fixed binary operating point used for target-lesion detection.

This splitting procedure allowed quantitative evaluation of the performance of the automatically generated segmentations. In the malignant subgroup, the diameter and volume of the TF-ROIs and GTs were evaluated on both GBCA and vCE images.

The diameter was defined as the maximum Euclidean distance between lesion voxels in physical space, calculated using the voxel spacing derived from the affine transformation matrix.

Additionally, to quantitatively evaluate the differences between the GT and TF-ROI segmentations, for the malignant lesion subgroup, the Dice score, the Hausdorff distance, and the differences in lesion diameter (Diff_d_) and volume (Diff_Vol_) and between the GT and TF-ROI segmentations were calculated separately for GBCA images (Dice_GBCA_, Hausdorff_GBCA_, Diff_d-GBCA_, Diff_Vol-GBCA_) and vCE images (Dice_vCE_, Hausdorff_vCE_, Diff_d-vCE_, Diff_Vol-vCE_). For all test set examinations, the NT-ROI volume was also evaluated by summing the volumes of the regions segmented in the whole volume.

MAMA-MIA nnU-Net’s detection rate of mass lesions/NMEs in agreement with the GT segmentation was evaluated on both vCE and GBCA volumes using the Dice score. Overlap between the TF-ROI and GT segmentations—defined as any case with Dice score > 0—was considered a correct detection of a mass lesion or NME by the model. The detection rate was calculated by dividing the number of correct detections by the number of GT segmentations.

### 2.5. Statistical Evaluation

Statistical analyses were performed using SigmaPlot software (version 15.0). The Shapiro–Wilk test was used to test for normality. Differences in the central tendency of the Diff_Vol_, Diff_d_, Dice, and Hausdorff scores were evaluated with paired *t*-tests for normally distributed data and with Wilcoxon signed-rank tests for non-normally distributed data. Differences in detection rates of the AI model between vCE and GBCA images were evaluated using the McNemar test. Detection rates are additionally reported with 95% confidence intervals calculated using the Wilson method. For continuous non-normally distributed metrics reported as medians, including Dice scores, Hausdorff distances, lesion diameter and volume differences, and segmentation volumes, 95% confidence intervals for the median were calculated using bootstrap resampling. The Alpha level was set to 0.05 with lower *p* being considered statistically significant for all tests. For the exploratory image-domain analysis, paired differences in normalized lesion intensity between DCE and vCE images were assessed using the Wilcoxon signed-rank test.

## 3. Results

The independent holdout test set included *n* = 256 breast MRI examinations, of which *n* = 75 had a histopathologically verified malignant finding. Six cases were excluded, as the patients were undergoing neoadjuvant chemotherapy, with visually minimal or no residual findings reported in the clinical radiologist’s report. Thus, the final independent holdout test dataset consisted of *n* = 69 malignant cases (mean age: 54.31 ± 11.45 years)—*n* = 40 mass-enhanced (ME) cases and *n* = 29 NME cases—lesion sizes ranging from 8 × 5 mm (3 mm foci) to 12 × 11 × 7 cm, and *n* = 181 benign cases (mean age: 52.32 ± 13.00 years) for analysis.

### 3.1. GAN Performance Metrics of vCE

The quantitative evaluation of the GAN-generated vCE images is presented in Table 2. SSIM, reflecting perceptual similarity, was 84.15 ± 3.78. PSNR, quantifying image reconstruction quality, was measured as 22.75 ± 1.57 dB. NRMSE, indicating the pixel-wise reconstruction error, reached 0.10 ± 0.01. Finally, MEDSYMAC, assessing symmetrical image accuracy, was measured as 0.009 ± 0.06.

#### Exploratory Image-Domain Characterization of DCE and vCE Images

**Figure 2:**
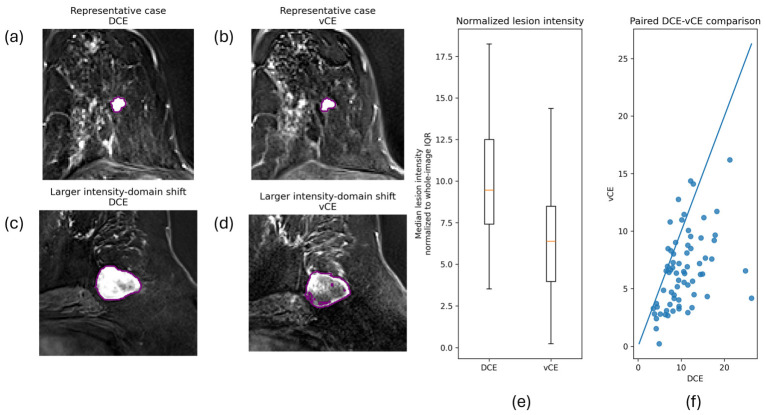
Exploratory image-domain characterization of GBCA-enhanced DCE and GAN-generated virtual contrast-enhanced breast MRI. A 44-year-old patient with a malignant enhancing lesion in the right breast, initially assessed as BI-RADS 4, is shown as a representative case with a paired normalized lesion-intensity difference close to the cohort median on GBCA-enhanced DCE MRI (**a**) and corresponding virtual contrast-enhanced (vCE) MRI (**b**). A 60-year-old patient with left axillary lymph-node metastases and suspicious left-breast non-mass enhancement, assessed as BI-RADS 6, is shown as an example with a larger intensity-domain shift between DCE (**c**) and vCE (**d**) images. Manual ground-truth lesion contours are shown in purple in panels (**a**–**d**). Box plots show normalized median lesion intensity within manual lesion masks for DCE and vCE images (**e**). The scatter plot shows paired normalized lesion-intensity values for DCE and vCE images (**f**). Lesion intensity was normalized to the whole-image interquartile range to account for differences in absolute image scaling between acquired and generated images.

Exploratory lesion-level intensity analysis was performed using paired measurements for all 69 malignant cases. Normalized median lesion intensity was higher on DCE images than on vCE images, with a median of 9.46 (interquartile range (IQR): 7.40–12.50) for DCE and 6.38 (IQR: 3.96–8.48) for vCE. The median paired difference between vCE and DCE was −3.67 (IQR: −6.03 to −0.95; *p* < 0.001). Representative paired examples are shown in Figure 2, including one case with a difference close to the cohort median and one case with a larger intensity-domain shift. These findings support the presence of measurable image-domain differences between GBCA-enhanced and vCE images despite visually similar lesion depiction.

### 3.2. Segmentation Performance of MAMA-MIA nnU-Net on GBCA-Enhanced and vCE Breast MRI

**Figure 3:**
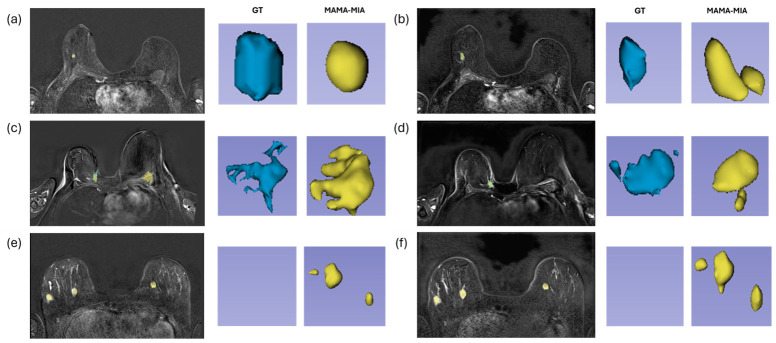
A 66-year-old patient with a malignant lesion in the right breast (BI-RADS 6), showing similar lesion delineation on both gadolinium-based contrast agent (GBCA)-enhanced (**a**) and virtual contrast-enhanced (vCE) images (**b**). A 76-year-old patient with a malignant lesion in the right breast (BI-RADS 5), illustrating comparable lesion segmentation in GBCA (**c**) and vCE (**d**); however, with the MAMA-MIA nnU-Nnet wrongly segmenting the additive pectoralis muscle on the left side as a tumor. A 33-year-old patient with benign lesions in both breasts (BI-RADS 2), highlighting segmentation similarity between GBCA (**e**) and vCE (**f**) images. Over-segmentation in both the first and second patient is clearly visible. Ground-truth (GT) segmentations are shown in blue, MAMA-MIA nnU-Net segmentations are shown in yellow, and the overlap between these two areas are shown in blue.

Figure 3 illustrates segmentation results from representative breast MRI cases. Visually, the lesion segmentation quality and alignment were frequently comparable between vCE and GBCA images, although discrepancies in segmentation boundaries were observable. These boundary differences are particularly evident in the 3D renderings, which reveal variations in segmentation morphology and lesion size. Additionally, one case (Figure 3c) shows over-segmentation by MAMA-MIA, where normal tissue in the contralateral breast is incorrectly segmented in the dynamic contrast-enhanced (DCE) image. In the benign case (third row), the model segments benign tissue, representing false-positive findings, as benign lesions were not intended for segmentation based on MAMA-MIA dataset training criteria.

Manual GT_GBCA_ segmentations were performed in all *n* = 69 malignant cases in the independent holdout test set. The median diameter was 22.2 mm (IQR: 18.3–34.4 mm; 95% confidence interval (CI): 19.8–27.3 mm) for segmented mass lesions and 36.1 mm (IQR: 21.8–55.4 mm; 95% CI: 24.1–48.0 mm) for NMEs. The median lesion volume for GT_GBCA_ was 1958.0 mm^3^ (interquartile range (IQR): 938.0–6368.5 mm^3^; 95% CI: 1185.0–2990.0 mm^3^).

The TF-ROI_GBCA_ segmentations automatically generated by MAMA-MIA had a median diameter of 24.4 mm (IQR: 16.4–36.5 mm; 95% CI: 20.4–29.1 mm), which was significantly different from the GT segmentation diameter (*p* ≤ 0.001). TF-ROI_GBCA_ had a median volume of 2390.0 mm^3^ (IQR: 1009.0–6099.0 mm^3^; 95% CI: 1578.0–2892.0 mm^3^), which was not significantly different than that for GT segmentation (*p* = 0.682).

The median GT_vCE_ lesion diameter was 27.9 mm (IQR: 17.2–43.6 mm; 95% CI: 22.9–33.0 mm) with a volume of 1607.0 mm^3^ (IQR: 604.5–5555.0 mm^3^; 95% CI: 1317.0–3751.0 mm^3^). Compared with GT segmentation, the median diameter of 26.3 mm (IQR: 12.7–36.0 mm; 95% CI: 18.0–29.6 mm) was significantly different (*p* ≤ 0.001) as was the median lesion volume of 2019.0 mm^3^ (IQR: 358.5–4406.5 mm^3^; 95% CI: 1095.0–2892.0 mm^3^) for TF-ROI_vCE_ segmentations (*p* = 0.048).

Comparison of segmentations revealed similar median Dice scores of 0.829 (IQR: 0.723–0.900; 95% CI: 0.786–0.865) for Dice_GBCA_, and 0.826 (IQR: 0.720–0.857; 95% CI: 0.770–0.836) for Dice_vCE_. Although this difference reached statistical difference (*p* < 0.001), the absolute median difference was small (0.003), indicating only a limited numerical difference in segmentation overlap among detected lesions. In contrast, the median Hausdorff_GBCA_ was 6.4 mm (IQR: 3.2–9.3 mm; 95% CI: 5.2–7.8 mm), while the median Hausdorff_vCE_ was 6.7 mm (IQR: 3.9–9.7 mm; 95% CI: 5.3–8.0 mm), with no statistically significant difference (*p* = 0.564).

**Figure 4:**
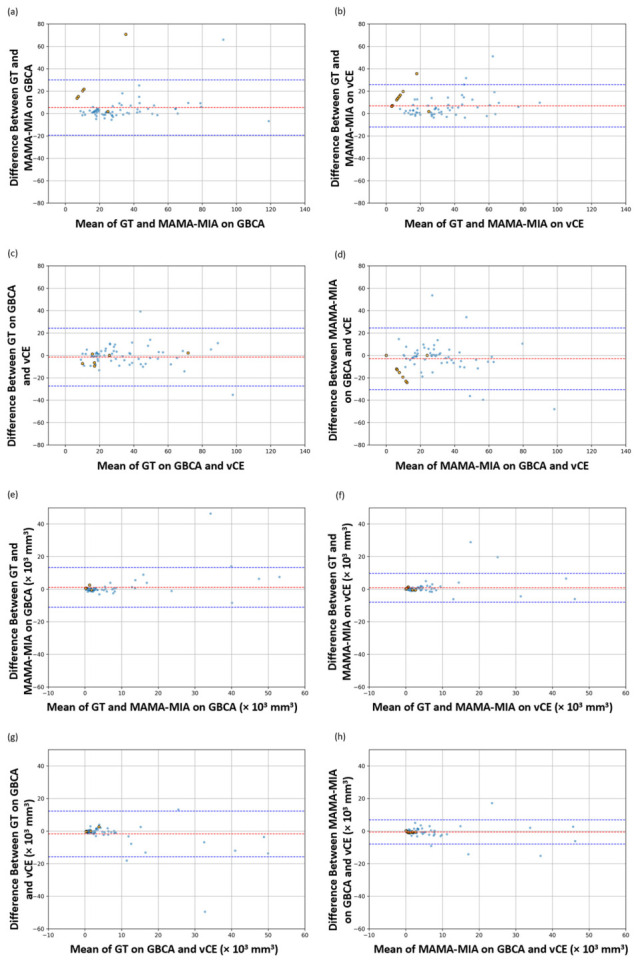
Bland–Altman analysis of segmentation diameter (mm) (**a**–**d**) and volume (mm^3^) (**e**–**h**) agreement between expert ground truth (GT) and MAMA-MIA nnU-Net segmentations for gadolinium-based contrast agent (GBCA)-enhanced and virtual contrast-enhanced (vCE) MRI images. Comparison of segmentation diameters between the GT_GBCA_ and TF-ROI_GBCA_ shows a median Diff_d-GBCA_ of 1.8 mm (IQR: −0.4–6.3 mm; 95% CI: 1.0–3.3 mm) (**a**–**d**). Similarly, a comparison between GT_vCE_ and TF-ROI_vCE_ shows a median Diff_d-vCE_ of 4.6 mm (IQR: 0.3–11.6 mm; 95% CI: 2.8–6.4 mm) which was significantly different (*p* = 0.014) from the Diff_d-GBCA_. Segmentation volume comparison between the GT_GBCA_ and TF-ROI_GBCA_ revealed a median Diff_Vol-GBCA_ of just 6.0 mm^3^ (IQR: −448.0–516.5 mm^3^; 95% CI: −138.0–165.0 mm^3^, (**e**–**h**)). The comparison of GT_vCE_ versus TF-ROI_vCE_ shows a median Diff_Vol-vCE_ of 137.0 mm^3^ (IQR: −400–991.5 mm^3^; 95% CI: 58.0–215.0 mm^3^) which was not significantly (*p* = 0.054) different from the Diff_Vol-GBCA_. Missed cases are shown in yellow and segmented cases are shown in blue. The red dashed horizontal line indicates the mean difference (bias), and the blue dashed horizontal lines indicate the 95% limits of agreement.

**Figure 5:**
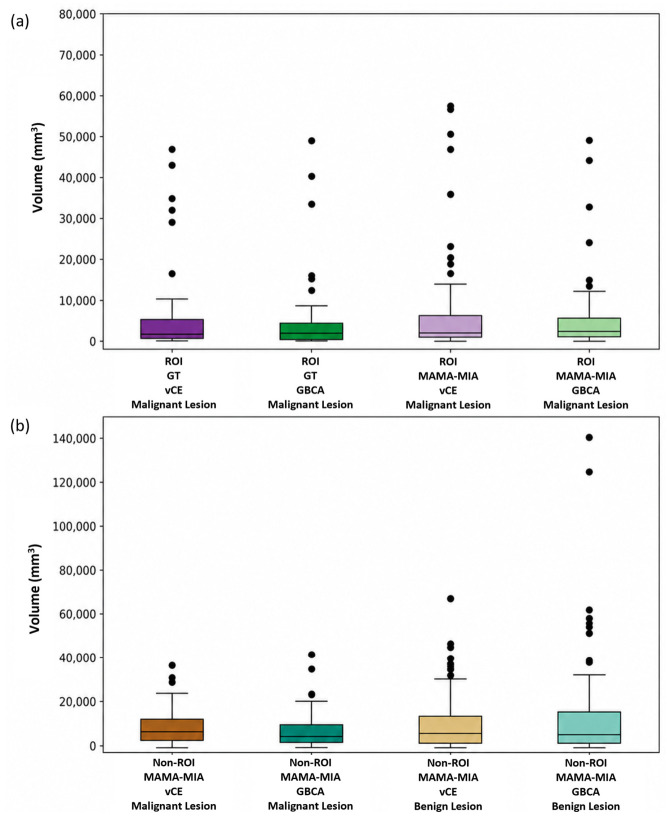
(**a**) Comparison of segmentation volumes (mm^3^) for malignant breast lesions between expert ground truth (GT) and MAMA-MIA nnU-Net on virtual contrast-enhanced (vCE) and gadolinium-based contrast agent (GBCA)-enhanced MRI images. (**b**) Comparison of total segmented breast volumes (mm^3^) for malignant cases of non-target regions of interest (NT-ROIs) and benign cases of NT-ROIs using MAMA-MIA nnU-Net across vCE and GBCA images.

Bland–Altman plots were used to compare the diameters and volumes of the lesions between GT and TF-ROI for both GBCA and vCE images (Figure 4). Figure 5a shows box plots of the segmentation volumes for malignant lesions, comparing GTs and TF-ROIs for both GBCA and vCE segmentations. In both the vCE and GBCA modalities, the automated method tended to produce slightly larger median lesion for TF-ROI volumes than GT segmentations.

### 3.3. Detection Ability of MAMA-MIA nnU-Net Model

MAMA-MIA nnU-Net successfully detected mass lesions and NMEs in 63 out of 69 malignant cases on GBCA-enhanced breast MRI, corresponding to a case-level detection sensitivity of 91% (95% CI: 82.3–96.0%). Automatic segmentation failed in six malignant cases (two NME and four ME), providing no TF-ROI segmentation. Among these missed cases: two patients presented with a DCIS (diameters: 70.6 and 25.7 mm), three with invasive breast cancer NST (20.2 mm; 13.6 mm; and 15.1 mm), and one case presented a pTis (21.7 mm). The total median lesion diameter was 21.0 mm (IQR: 14.8–37.0 mm). To characterize failure patterns, missed lesions were descriptively reviewed with regard to lesion morphology, histopathological subtype, and lesion diameter.

For the vCE breast MRI, MAMA-MIA nnU-Net correctly detected 58 out of 69 malignant lesions, corresponding to a case-level detection sensitivity of 84% (95% CI: 73.7–90.9%). In the remaining *n* = 11 cases (three NME and eight ME), the TF-ROIs were not segmented automatically. The characteristics comprised one minimal residual disease (MRM pTis Ris0; diameter: 21.7 mm), six cases of invasive breast cancer (15.2 mm; 13.6 mm;15.9 mm; 42.3 mm; 15.1 mm and 10.9 mm), one pTis of 21.8 mm, one case of invasive lobular carcinoma (ILC, 17.0 mm) and two DCIS (25.7 mm; 22.7 mm). These 11 lesions had a mean GT diameter of 16.5 ± 9.0 mm.

Overall, missed lesions were observed in both mass-enhancing and non-mass-enhancing lesions and across different histopathological subtypes, suggesting that detection failures were not confined to a single lesion morphology. Given the limited number of missed cases, no formal subgroup comparison was performed. The difference in case-level lesion detection sensitivity rate (mass lesion and NME) of the MAMA-MIA nnU-Net between vCE and GBCA was not statistically significant (*p* = 0.180) for this sample size.

**Figure 6:**
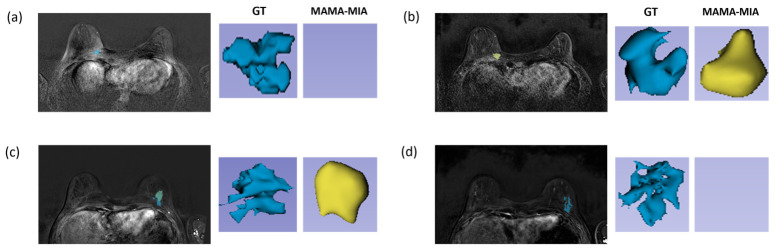
(**a**,**b**) A 52-year-old patient with a malignant lesion in the right breast (BI-RADS 6). The ground-truth (GT) segmentation can be seen in (**a**); however, MAMA-MIA nnU-Net failed to capture it on gadolinium-based contrast agent (GBCA)-enhanced images. In the virtual contrast-enhanced (vCE) images, the MAMA-MIA nnU-Net captures the lesion (**b**). (**c**,**d**) A 41-year-old patient with a malignant lesion in the left breast (BI-RADS 6), which is visible in GBCA (**c**), while on the vCE image (**d**), the MAMA-MIA segmentation was unsuccessful in segmenting it. GT segmentations are shown in blue, and MAMA-MIA nnU-Net segmentations are shown in yellow.

Interestingly, the overlap of non-segmented TF_GBCA/VCE_ lesions was not complete: one-third (2/6) of the lesions missed on the GBCA images were correctly detected by the MAMA-MIA nnU-Net on the vCE images. Figure 6 shows examples where MAMA-MIA failed to capture lesions in GBCA (a) and vCE (b) images.

#### Non-Target-Area Segmentation by MAMA-MIA nnU-Net on GBCA-Enhanced and vCE Breast MRI

Excess segmentation of the non-target volume by MAMA-MIA nnU-Net was observed for both GBCA and vCE breast MR images. For NT-ROI_GBCA_, the median excess volume was 5754.0 mm^3^ (IQR: 3094.5–12,722.0 mm^3^; 95% CI: 4611.5–7656.5 mm^3^), whereas NT-ROI_vCE_ showed a slightly higher median of 6072.0 mm^3^ (IQR: 2275.0–11,386.0 mm^3^; 95% CI: 4562.5–7594.5 mm^3^). No significant differences in NT-ROI volume were observed in either the malignant (*p* = 0.069) or benign (*p* = 0.608) groups. Figure 5b compares NT-ROI volumes for malignant cases on both vCE and GBCA images, alongside benign breast volumes for comparison. The median total volumes for malignant cases were slightly lower than those for benign cases. No statistical differences were observed between the benign and malignant volumes for NT-ROI_vCE_ (*p* = 0.693) or NT-ROI_GBCA_ (*p* = 0.533).

## 4. Discussion

This work evaluated the generalizability of MAMA-MIA nnU-Net, originally trained on GBCA-enhanced breast MRI acquisitions, to an external dataset that additionally comprised GAN-derived virtual contrast-enhanced (vCE) images generated from an unenhanced breast-MRI protocol.

Our results demonstrated a detection rate of 91% (95% CI: 82.3–96.0%) for MAMA-MIA nnU-Net on GBCA-enhanced breast MRI and an unexpected robustness on vCE images, albeit with a lower detection rate, of 84% (95% CI: 73.7–90.9%). Diameter and volume differences between MAMA-MIA segmentations and the manual ground truth were not significantly different between vCE and GBCA-enhanced images.

Surprisingly, one-third (2/6) of the lesions missed by MAMA-MIA nnU-Net on the GBCA-enhanced breast MR images were detected in the vCE-generated segmentations. 

vCE techniques have recently been introduced in various medical imaging applications, including breast MRI [18,33]. Although still in its technical infancy, the approach has gained significant scientific interest, partly driven by the aim of facilitating non-contrast-enhanced breast MRI, for example, in supplemental breast cancer screening for women with dense breast tissue on X-ray mammography. Non-contrast-enhanced breast MRI approaches may support efforts to increase the accessibility of supplemental imaging methods by reducing both the cost and effort associated with GBCA administration, while increasing the sensitivity for breast cancer detection compared with X-ray mammography itself [12,17,34]. Initial studies using vCE images generated from T1- and T2-weighted acquisitions demonstrated limited results. These outcomes improved significantly when multiple-b-value DWI, including an ultra-high b-value of 1500 s/mm^2^, was added to the training of the neural networks, as suggested by Liebert et al. [35]. This protocol was also included in the datasets used in our study.

Network architectures used in studies on virtual contrast enhancement include U-Nets (e.g., in breast MRI) [35], GANs (in prostate MRI) [23], and, more recently, diffusion models (e.g., in spine MRI) [36], all aiming to improve image quality and the conspicuity of pathologies. The structural similarity index achieved by the GAN in our study was comparable to values reported for breast and brain (0.84–0.88) [18,37], although its calculation over the full breast in the literature may limit its clinical diagnostic relevance as an indicator of performance of the networks. For breast MRI, the added value of vCE approaches over high-quality non-contrast breast-MRI protocols has not yet been proven. It remains to be established whether it can significantly improve diagnostic accuracy and reader confidence over conventional non-contrast-enhanced breast MRI [14,38].

In parallel with image-to-image contrast transformation (as in vCE), AI is increasingly being used to support lesion detection in breast imaging. While AI is increasingly clinically adopted in X-ray mammography [39], its application in GBCA-enhanced breast MRI is still largely limited to research. AI has the potential to reduce reading time, improve accuracy, and assist in triaging breast MRI examinations. The MAMA-MIA nnU-Net model used in our study [24] exemplifies the potential for the collaborative development of robust, transparent, and generalizable AI in breast MRI.

Our findings indicate an unexpected, though limited, generalizability of a GBCA-trained AI to vCE images derived from non-contrast images that inherently lack the physiological ground-truth properties of GBCA-enhanced acquisitions. This is reflected in a statistically significant but numerically small decrease in the median Dice coefficient, from 0.829 (IQR: 0.723–0.900; 95% CI: 0.786–0.865) for GBCA-enhanced images to 0.826 (IQR: 0.720–0.857; 95% CI: 0.770–0.836)) for vCE images, compared with the mean value of 0.76 reported for full-image tumor segmentation by MAMA-MIA [24]. This finding is noteworthy, as vCE images would typically represent an out-of-distribution (OOD) scenario for an AI trained exclusively on GBCA-enhanced MRI datasets. Such OOD conditions usually result in a marked drop in performance.

The added exploratory image-domain analysis demonstrated that normalized lesion intensity differed between GBCA-enhanced DCE and vCE images, indicating that the vCE domain was not intensity-equivalent to the acquired GBCA-enhanced domain. Therefore, the observed model performance should not be interpreted as evidence of domain equivalence. Rather, the findings suggest partial cross-domain transferability of the GBCA-trained MAMA-MIA nnU-Net to vCE images, despite measurable differences in relative lesion intensity and image contrast.

A related consideration is that technical similarity between generated and acquired images does not necessarily imply preservation of all diagnostically relevant features. Although SSIM, PSNR, NRMSE, and MEDSYMAC provide useful global measures of image similarity, it is important to emphasize that those metrics do not directly validate lesion morphology or enhancement patterns in a manner that allows drawing any assumptions about the clinical utility or applicability of such an approach.

Whilst the overall image quality of the vCE images was considered high, our data evaluation also demonstrated that lesion enhancement itself was significantly lower in the vCE images, which matches the observed diagnostically relevant, although statistically nonsignificant, reduction in detection rate of malignant lesions in the vCE cohort.

Still, the vCE images seemingly retained sufficient lesion-related information for partial AI-based lesion detection, despite the measurable image-domain differences, The ability to “fool” both the human eye and AI is particularly interesting given the different biophysical origins of the “enhancement” information in both approaches. GBCA-enhanced images reflect true GBCA-distribution information arising from altered perfusion, microvascular structures, and alterations in tissue permeability. In contrast, vCE approaches seem to approximate this information in a rather indirect and correlative manner, at least in part, from the multi-b-value DWI, as described by Liebert et al. [35]. Indeed, DWI parameters have been suggested to be correlated partially with microvascular density [40,41] aside of intravoxel incoherent motion (IVIM). Yet, the information in vCE images as generated by the GAN more likely reflects a spectrum of correlating tissue characteristics, each partially associated with biophysical properties and also influencing the enhancement characteristics after GBCA administration and vCE images should thus not be interpreted as being directly equivalent to the perfusion-based biophysiological processes observed in GBCA-enhanced imaging. Rather, they represents a broader and more limited correlation.

Our study has several limitations: The in-house dataset was relatively small, comprising *n* = 2126 cases—including *n* = 256 cases in the independent holdout test dataset—originating from a single hospital. However, as our site is not part of the MAMA-MIA consortium, the data represent a fully independent third-party breast MRI dataset. Since all scans were acquired using a single MR vendor and field strength (3T, Siemens Healthineers), our findings cannot be generalized to other field strengths, devices, or vastly different MR acquisition protocols. Moreover, we focused on malignant lesion detection rates and segmentation performance at the fixed binary operating point of the MAMA-MIA nnU-Net rather than conducting an in-depth analysis of diagnostic accuracy. Accordingly, additional “false-positive” areas segmented by MAMA-MIA nnU-Net were only described quantitatively and not assessed further. Thus, we did not evaluate the algorithm’s clinical applicability or diagnostic capabilities. Finally, we did not investigate different network architectures for generating vCE images from the in-house dataset or their potential impact on the performance of MAMA-MIA nnU-Net. From a workflow perspective, clinical implementation would also require robust automation of sequence identification, preprocessing, vCE generation, segmentation inference, and quality control, as failures at any of these steps may propagate into the final segmentation output. Although the training/validation and independent holdout test sets were separated at the patient level, the GAN-based vCE model was developed using single-site, single-vendor internal data. Therefore, limited generalizability to different scanner vendors, field strengths, acquisition protocols, or institutional imaging characteristics remains possible and requires prospective multi-center validation. Another technical limitation is related to the heterogeneous slice thickness of the acquired input sequences. While the T1w and GBCA-enhanced DCE images were acquired with thinner slices, T2w and DWI were acquired with 4 mm slice thickness and therefore required through-plane alignment/resampling for paired vCE generation. This preprocessing enables spatial correspondence between input and target images but cannot recover through-plane information that was not present in the original lower-resolution acquisitions. Consistent with the prior breast DWI literature showing that a 4 mm section thickness may result in relevant partial-volume effects, particularly for small lesions, reduced through-plane detail in the T2w/DWI inputs may have contributed to residual differences between GAN-generated vCE and GBCA-enhanced images, including lesion-boundary sharpness and downstream segmentation behavior [42]. The independent effect of slice-thickness mismatch was not assessed in the present study. Finally, it needs to be emphasized here that no clinical implications can be drawn from the preliminary data and further research is necessary before clinical meaningful conclusions with regard to the applicability of vCE in breast MRI and its analysis with AI applications can be drawn.

Future research should therefore prioritize: (1) prospective multi-center validation across different scanner vendors, field strengths, and acquisition protocols; (2) systematic evaluation of GAN-related artifacts and their propagation into downstream segmentation errors; and (3) exploration of alternative generative architectures and their impact on AI model behavior.

## 5. Conclusions

We used an nnU-Net model trained on external GBCA-enhanced breast-MRI data to segment lesions in both GBCA-enhanced and vCE breast MR images, demonstrating that lesion detection in vCE images is technically feasible, albeit with reduced performance metrics. These preliminary findings indicate the need for further research in this area to critically assess the potential generalizability of AI applications to vCE in breast MRI.

## Figures and Tables

**Table 1:** MRI protocol of the in-house breast MRI examinations.

Sequence	Sequence Type	Matrix Size	FoV (mm × mm)	Slice Thickness (mm)	TR (ms)	TE (ms)	IR (ms)	Fat Saturation
T1w ^1^	3D-GRE	448 × 448 × 112–128	360 × 360–430 × 430	1.5–1.8	5.97	2.46	-	None
T2w	2D-SE	448 × 448 × 34–49	340 × 340–430 × 430	4	3570–5020	60, 70	230	STIR
DWI ^2^	2D-IR-DWI-EPI	256 × 160–200 × 34–49	350 × 219–430 × 269	4	6290–9660	66, 70	220, 250	STIR

FoV = Field of view, T1w = T1-weighted, T2w = T2-weighted, TR = repetition time, TE = echo time, IR = inversion recovery time, DWI = diffusion-weighted imaging, GRE = gradient echo, SE = spin echo, EPI = echo-planar imaging, GBCA = gadolinium-based contrast agent, STIR = short-tau inversion recovery. ^1^ Acquired before and during the five time-points after intravenous GBCA injection (gadobutrol; Bayer, Leverkusen, Germany; 0.1 mmol/kg/body weight, injection speed = 2 mL/s). ^2^ DWI scans were acquired using three b-values: 50, 750, and 1500 s/mm^2^.

**Table 2:** GAN performance metrics of vCE.

SSIM	PSNR [dB]	NRMSE	MEDSYMAC
84.15 ± 3.78	22.75 ± 1.57	0.10 ± 0.01	0.009 ± 0.06

SSIM = Structural similarity index measure; PSNR = peak signal-to-noise ratio; NRMSE = normalized root mean square error; MEDSYMAC = median symmetrical accuracy; GBCA = gadolinium-based contrast agent; vCE = virtual contrast-enhanced. Table shows mean ± standard deviation of each matrix. Qualitative expert assessment of GAN-generated vCE images was available for all 69 malignant cases. The median rating was 5 (IQR: 4–5), with a mean of 4.43 ± 0.67. Overall, 62/69 cases (89.9%) were rated 4 or 5, while no case was rated 1 or 2.

## Data Availability

The Erlangen breast MRI datasets originate from clinical routine, protected by national privacy laws, and hence are not publicly available or shareable with third parties due to the retrospective nature of the study performed and the associated IRB approval for the study limiting the data use. The additive external and publicly available datasets (at the time of the study) used in the study are the following, with the detailed regulatory and licensing information to be found under the respective source. The external datasets and their (future) availability and conditions of availability are not under the control of the authors of this study. At the time the study was conducted, the accessibility was as follows: The I-SPY1/ACRIN 6657 trial (I-SPY1) [26], breast MRI datasets were accessible (at the time of the study) via The Cancer Imaging Archive (TCIA) [25]. The dataset was accessed via the Cancer Imaging Archive homepage. The data was accessible at the time of the study under the CC BY 3.0 license. The I-SPY2/ACRIN 6698 trial (I-SPY2) [27] breast MRI datasets were accessible (at the time of the study) via The Cancer Imaging Archive (TCIA) [25]. The dataset was accessed via the Cancer Imaging Archive homepage. The data was accessible at the time of the study under the CC BY 4.0 license. The NACT-Pilot trial (NACT) [28] breast MRI datasets were accessible (at the time of the study) via The Cancer Imaging Archive (TCIA) [25]. The dataset was accessed via the Cancer Imaging Archive homepage. The data was accessible at the time of the study under the CC BY 3.0 license. The Duke-Breast-Cancer-MRI (DUKE) [29] breast MRI datasets were accessible (at the time of the study) via The Cancer Imaging Archive (TCIA) [25]. The dataset was accessed via the Cancer Imaging Archive homepage. The data was accessible at the time of the study under the CC BY-NC 4.0 license. The source data of the figures are provided. The source data are provided in this paper.

## Abbreviations

The following abbreviations are used in this manuscript:

AI	Artificial intelligence
CI	Confidence interval
DCE	Dynamic contrast-enhanced
Dice_GBCA_	Dice difference between GT segmentations and GBCA MAMA-MIA segmentations
Dice_vCE_	Dice difference between GT segmentations and vCE MAMA-MIA segmentations
Diff_d-GBCA_	The differences in diameter for GBCA segmentations
Diff_d-vCE_	The differences in diameter for vCE segmentations
Diff_Vol-GBCA_	The differences in volume for GBCA
Diff_Vol-vCE_	The differences in volume for vCE
DWI	Diffusion-weighted imaging
GAN	Generative adversarial network
GBCA	Gadolinium-based contrast agent
GT_GBCA_	Ground truth of target lesion for GBCA
GT_vCE_	Ground truth of target lesion for vCE
Hausdorff_GBCA_	Hausdorff distance between GT segmentations and GBCA MAMA-MIA segmentations
Hausdorff_vCE_	Hausdorff distance between GT segmentations and vCE MAMA-MIA segmentations
IQR	Interquartile range
MEDSYMAC	Median symmetrical accuracy
MRI	Magnetic resonance imaging
NRMSE	Normalized root mean square error
NT-ROI _GBCA_	Non-target regions of interest of any segmented area besides the primary lesion in GBCA
NT-ROI_vCE_	Non-target regions of interest of any segmented area besides the primary lesion in vCE
PSNR	Peak signal-to-noise ratio
SSIM	Structural similarity index measure
TF-ROI_GBCA_	Target-finding regions of interest of the lesions in GBCA
TF-ROI_vCE_	Target-finding regions of interest of the lesions in vCE
vCE	Virtual contrast-enhanced

## Appendix A

**Table A1:** Overview of previous studies partially including data from the same internal dataset.

Authors	Title	Publication	Year
George, A., et al. [19]	“U-Net and GAN for Virtual Contrast in Breast MRI”	Bildverarbeitung für die Medizin	2025
Schreiter, H., et al. [20]	“Virtual Dynamic Contrast Enhanced Breast MRI Using 2D U-Net Architectures”	Artificial Intelligence and Imaging for Diagnostic and Treatment Challenges in Breast Care	2025
Liebert, A., et al. [43]	“Feasibility to virtually generate T2 fat-saturated breast MRI by convolutional neural networks”	European Radiology Experimental	2025
Liebert, A., et al. [35]	“Impact of non-contrast-enhanced imaging input sequences on the generation of virtual contrast-enhanced breast MRI scans using neural network”	European Radiology	2024
Liebert, A., et al. [44]	“Smart forecasting of artifacts in contrast-enhanced breast MRI before contrast agent administration”	European Radiology	2024
Brock, L., et al. [45]	“Comparative Study on Co-registration Techniques for Diffusion-Weighted Breast MRI and Improved ADC Mapping”	Biomedical Image Registration	2024
Kapsner, L.A., et al. [46]	“Lesion-conditioning of synthetic MRI-derived subtraction-MIPs of the breast using a latent diffusion model”	Scientific Reports	2024
Kapsner, L.A., et al. [47]	“Prevalence and influencing factors for artifact development in breast MRI-derived maximum intensity projections”	Acta Radiologica	2023
Kapsner, L.A., et al. [48]	“Image quality assessment using deep learning in high b-value diffusion-weighted breast MRI”	Scientific Reports	2023
Kapsner, L.A., et al. [49]	“Automated artifact detection in abbreviated dynamic contrast-enhanced (DCE) MRI-derived maximum intensity projections (MIPs) of the breast”	European Radiology	2022
